# Author Correction: Large size in aquatic tetrapods compensates for high drag caused by extreme body proportions

**DOI:** 10.1038/s42003-022-03458-x

**Published:** 2022-05-19

**Authors:** Susana Gutarra, Thomas L. Stubbs, Benjamin C. Moon, Colin Palmer, Michael J. Benton

**Affiliations:** 1 School of Earth Sciences, University of Bristol, Life Sciences Building, 24 Tyndall Avenue, Bristol, BS8 1TQ UK; 2 Natural History Museum, Cromwell Road, London, SW7 5BD UK

**Keywords:** Palaeontology, Palaeoecology, Biomechanics

Correction to: *Communications Biology* 10.1038/s42003-022-03322-y, published online 28 April 2022.

The wrong Supplementary Data 1 was originally published with this article; it has now been replaced with the correct version. The html version of this article has been corrected.

## Supplementary information

Updated Supplementary Data 1

