# Supplementary material for: Large size in aquatic tetrapods compensates for high drag caused by extreme body proportions
Source: Commun Biol. 2022 Apr 28;5:380. doi: 10.1038/s42003-022-03322-y (PMC9051157; doi:10.1038/s42003-022-03322-y)
Supplement: Supplementary file 2 — Supplementary Information [file 42003_2022_3322_MOESM2_ESM.pdf]

Supplementary Information for:

'Large size in aquatic tetrapods compensates for high drag caused by extreme body proportions'

Susana Gutarra, Thomas L. Stubbs, Benjamin C. Moon, Colin Palmer, Michael J. Benton

Content

Supplementary figures…………………………………………………………………… 2

Supplementary tables…………………………………………………………………….. 11

Supplementary methods…………………………………………………………………..17

NURBS modelling ……………………………………………………………….. 17

Sensitivity tests ……………………..…………………………………………..... 18

Composite sauropterygian tree…………………………………………………..... 18

Time scaling………………………………………………………………...…..19

Supplementary references……………………………………………………………...19

Supplementary figures

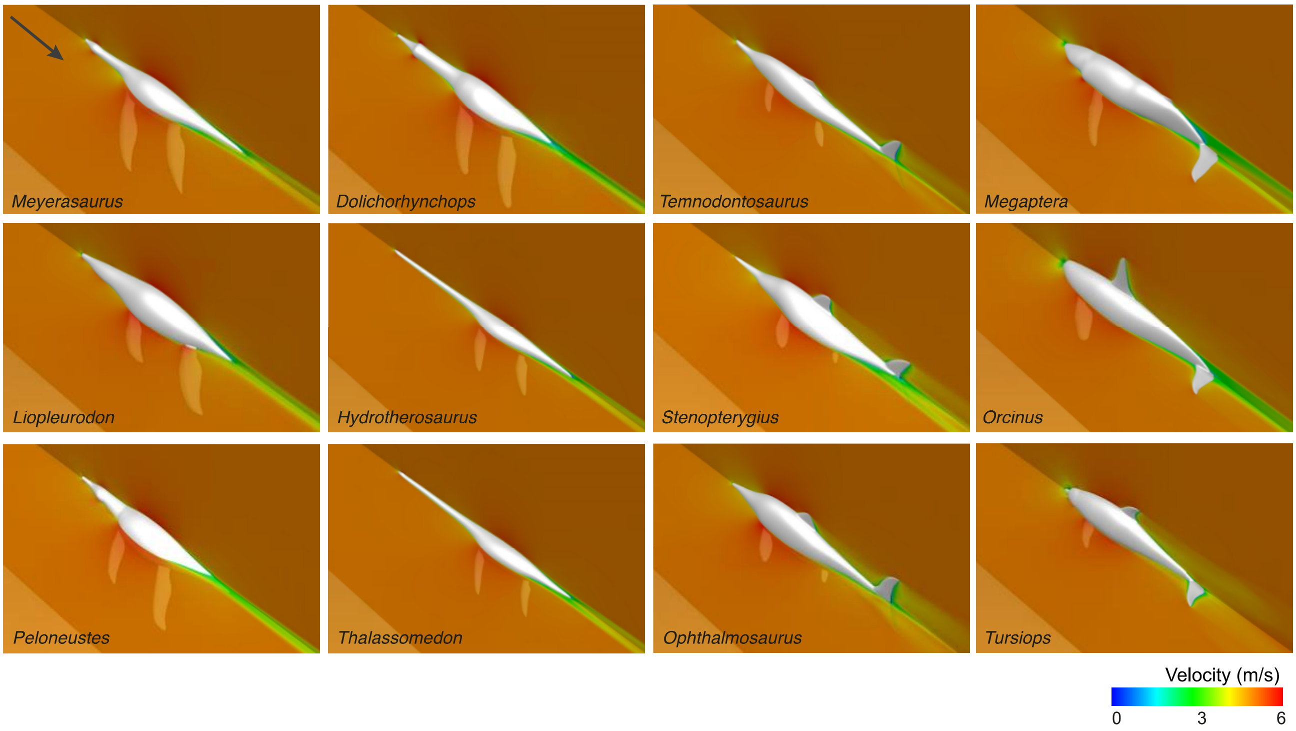

Supplementary Figure 1. Velocity plots of the flow around plesiosaurs, ichthyosaurs and modern cetaceans. Models are shown from the dorsal perspective, with two-dimensional velocity plots along the midsagittal (xz) and frontal (xy) planes for $Re = 5 \times 10^6$ (inlet velocity of 5 ms^{-1}). Arrow indicates the direction of flow.

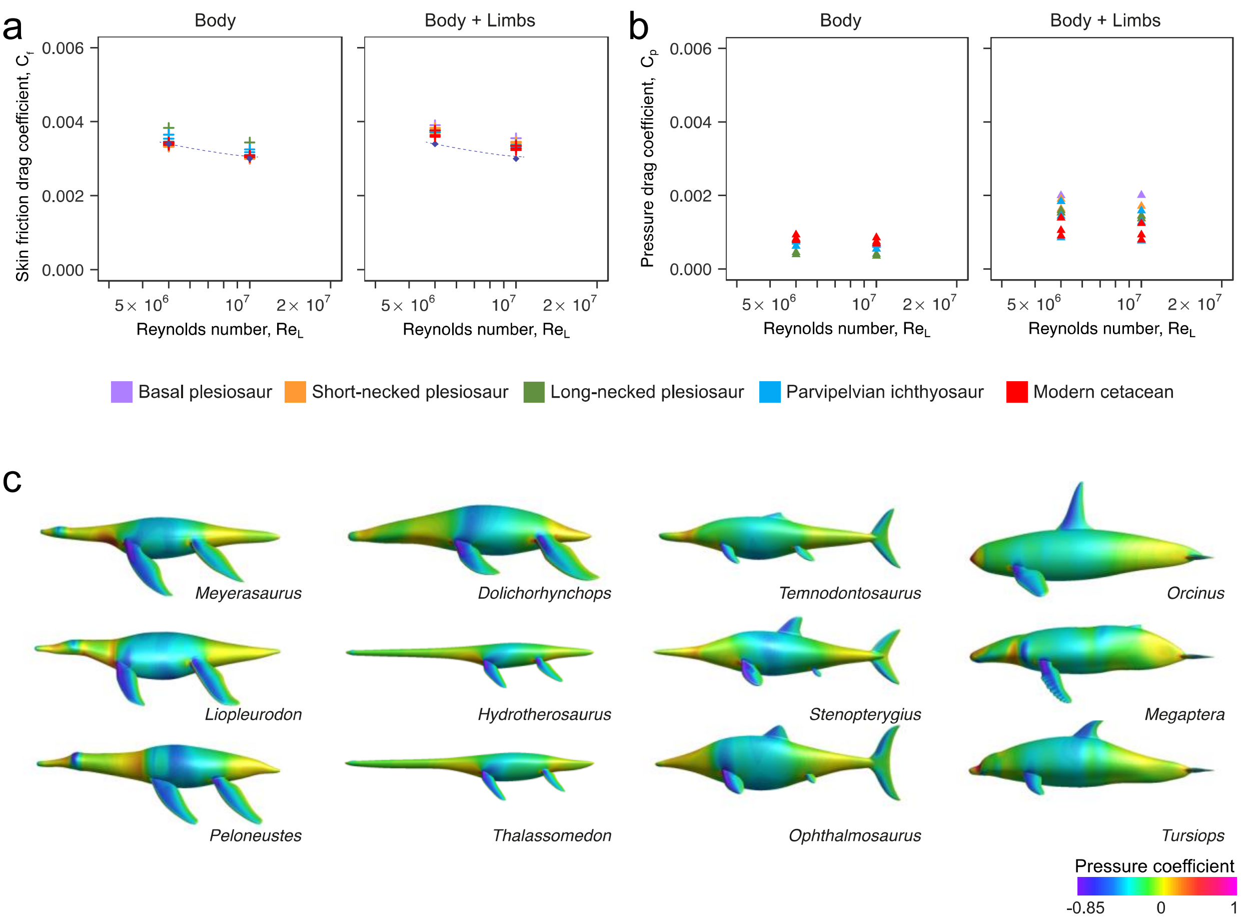

Supplementary Figure 2. Friction and pressure drag coefficient derived sauropterygians, ichthyosaurs and cetaceans. Computed frictional drag coefficient **(a)** and pressure drag coefficient **(b)** estimated for the full models including the limbs ('body', left) and the limbless models ('body + limbs', right) at two Reynolds numbers, 5×10^6 and 10^7. Colour scheme as in Figure 1a. **(c)** Three-dimensional contour plots of the pressure coefficient distribution for a Re = 5×10^6 (inlet velocity of 5 ms^{-1}).

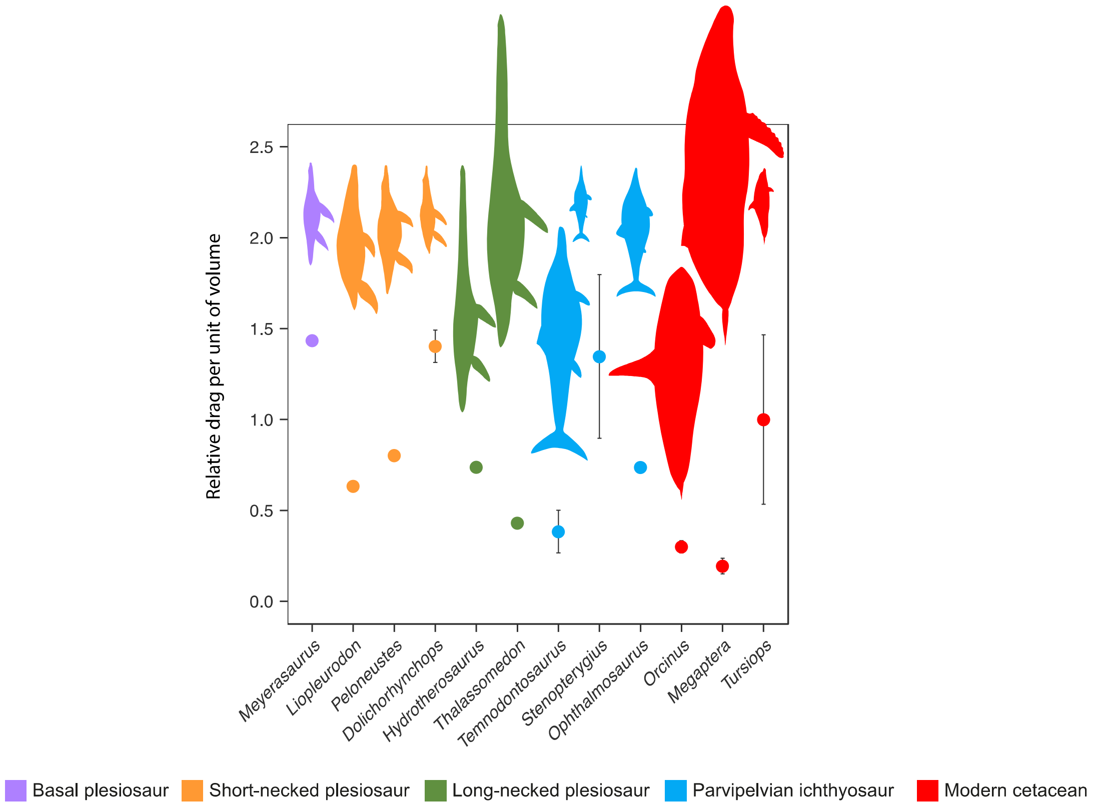

Supplementary Figure 3. Relative drag per unit of volume for life-size scaled models compared at the same inlet velocity of 2 ms^{-1}. Alternative analysis to the one depicted in Figure 2b with simulations performed at a different velocity. Error bars represent standard deviation accounting for interspecific size variation (see Supplementary Data). Colour scheme as in Figure 1a.

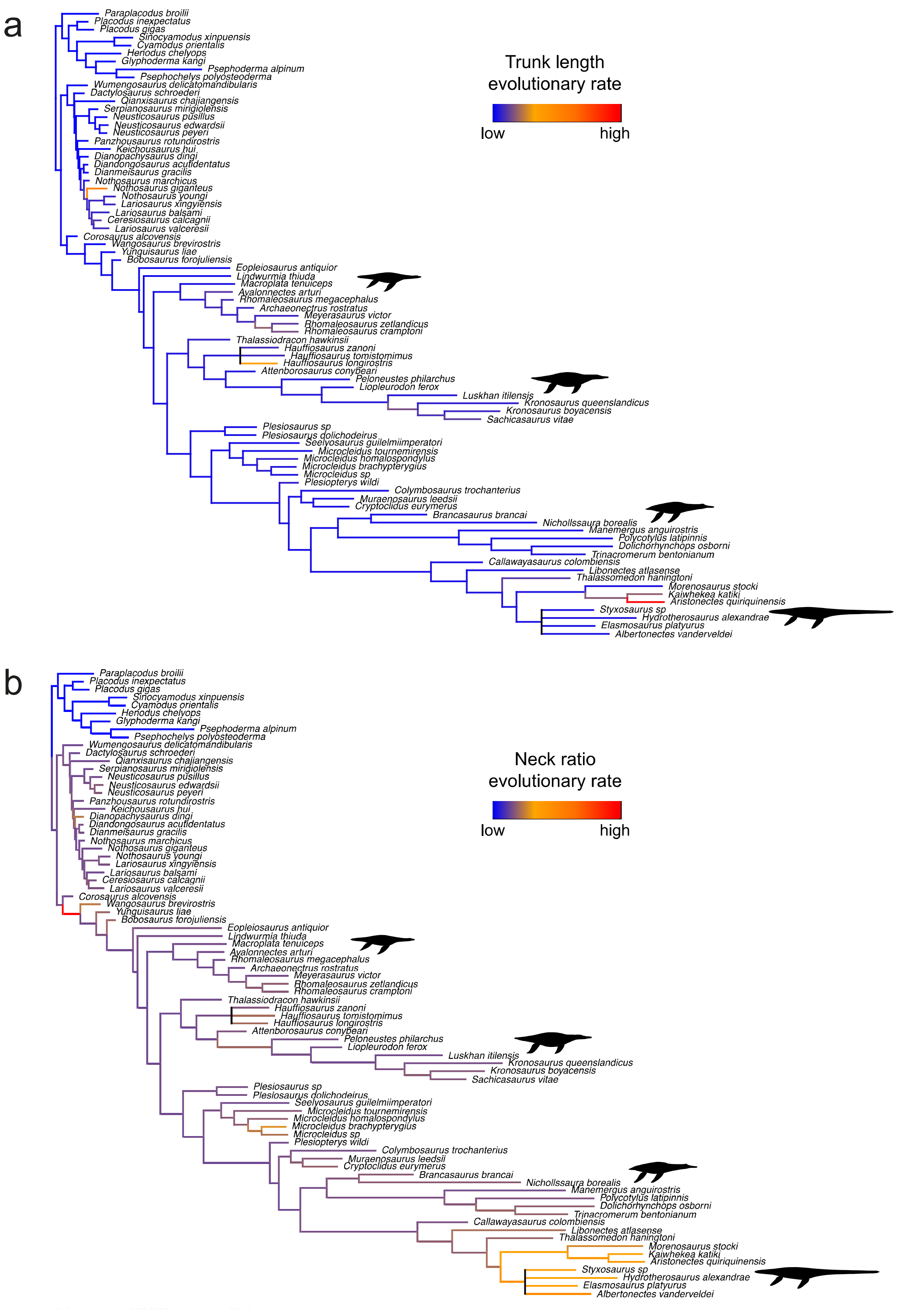

Supplementary Figure 4. Evolutionary rates for the trunk length and neck ratio in Sauropterygia using Hedman-scaled trees. Sauropterygian trees showing the evolutionary rates for trunk length **(a)** and neck ratio **(b)** represented by colour gradient. Average results from analyses on 20 Hedman-dated trees are shown. Rates are based on the mean scalar evolutionary rate parameter. Silhouettes represent (from top to bottom) basal plesiosaurs, Thalassophonea, Polycotylidae and Elasmosauridae.

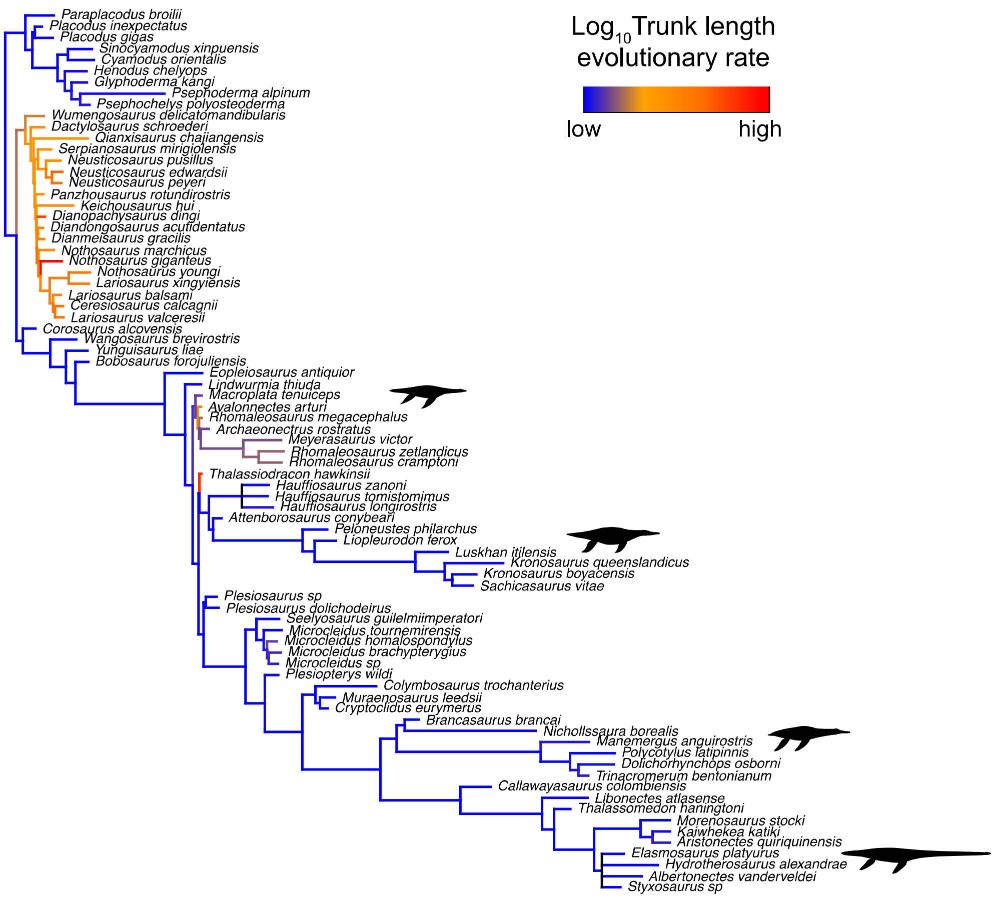

Supplementary Figure 5. Evolutionary rates for the Log$_{10}$-transformed trunk length in Sauropterygia using cal3-scaled trees. Sauropterygian phylogeny showing the average results of evolutionary rates analyses performed on 20 cal3-scaled trees. Rates are based on the mean scalar evolutionary rate parameter and are represented by colour gradient. Silhouettes represent (from top to bottom) basal plesiosaurs, Thalassophonea, Polycotylidae and Elasmosauridae.

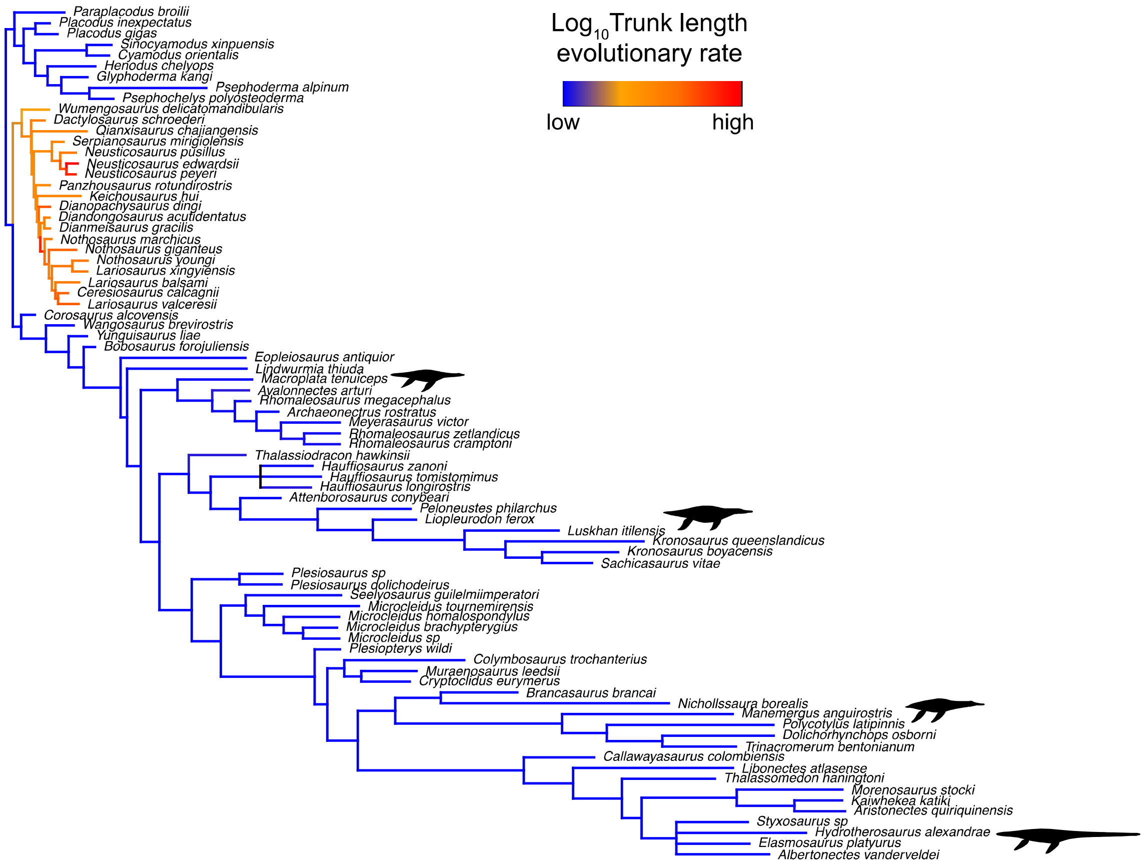

Supplementary Figure 6. Evolutionary rates for the Log$_{10}$-transformed trunk length in Sauropterygia using Hedman-scaled trees. Sauropterygian phylogeny showing the average results of evolutionary rates analyses performed on 20 Hedman-scaled trees. Rates are based on the mean scalar evolutionary rate parameter and are represented by colour gradient. Silhouettes represent (from top to bottom) basal plesiosaurs, Thalassophonea, Polycotylidae and Elasmosauridae.

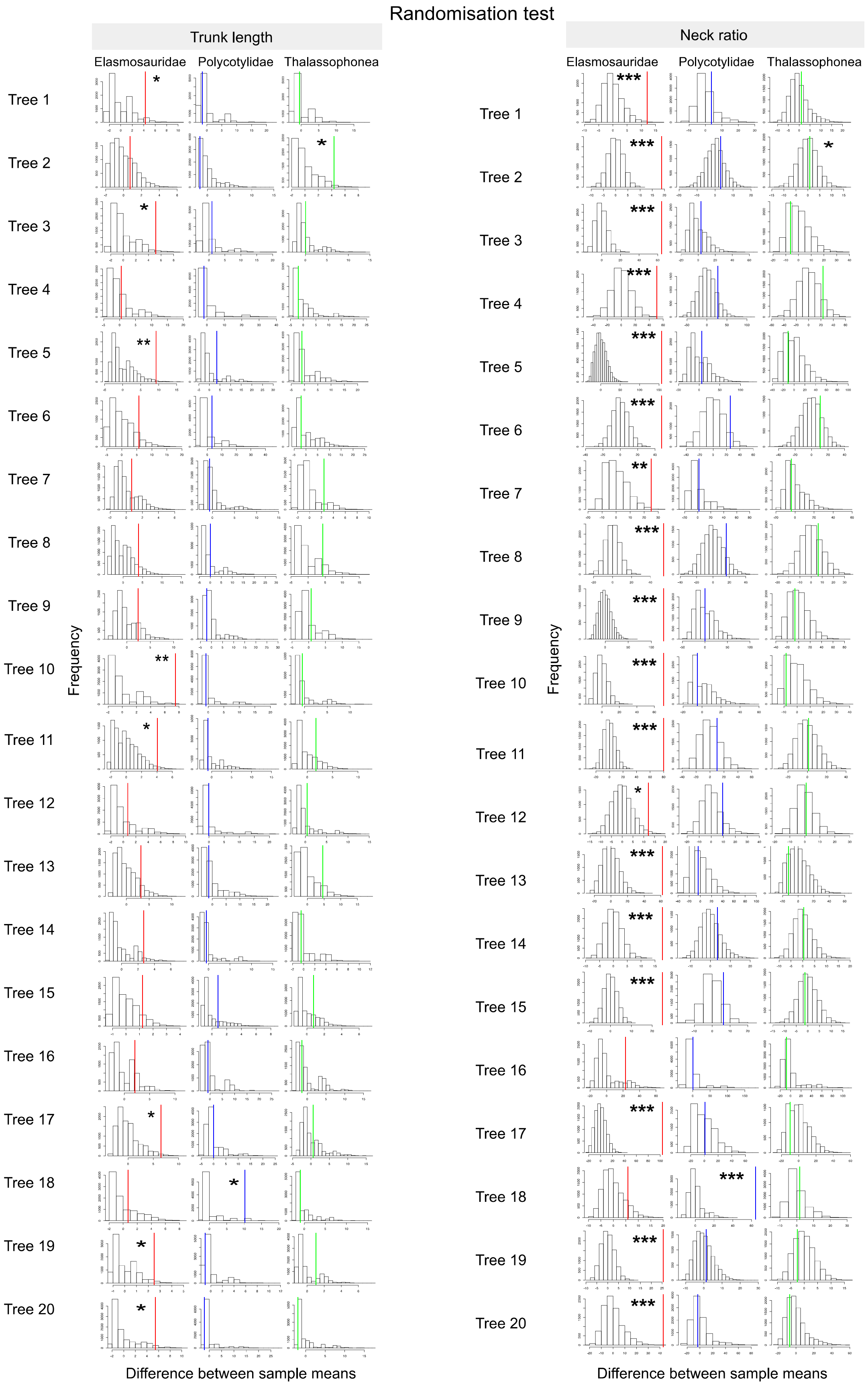

Supplementary Figure 7. Randomisation tests for the rates of evolution of trunk length and neck ratio. The differences between sampled mean evolutionary rates and the background evolutionary rates obtained from twenty cal3-dated phylogenies are shown for three clades of derived plesiosaurs: Elasmosauridae, Polycotylidae and Thalassophonea. Vertical bars indicate values for the specified clade against 9999 randomised samples. Asterisks represent statistical significance (* = *p*-value < 0.05; ** = *p*-value < 0.01; *** = *p*-value < 0.001; see also Supplementary Table 6).

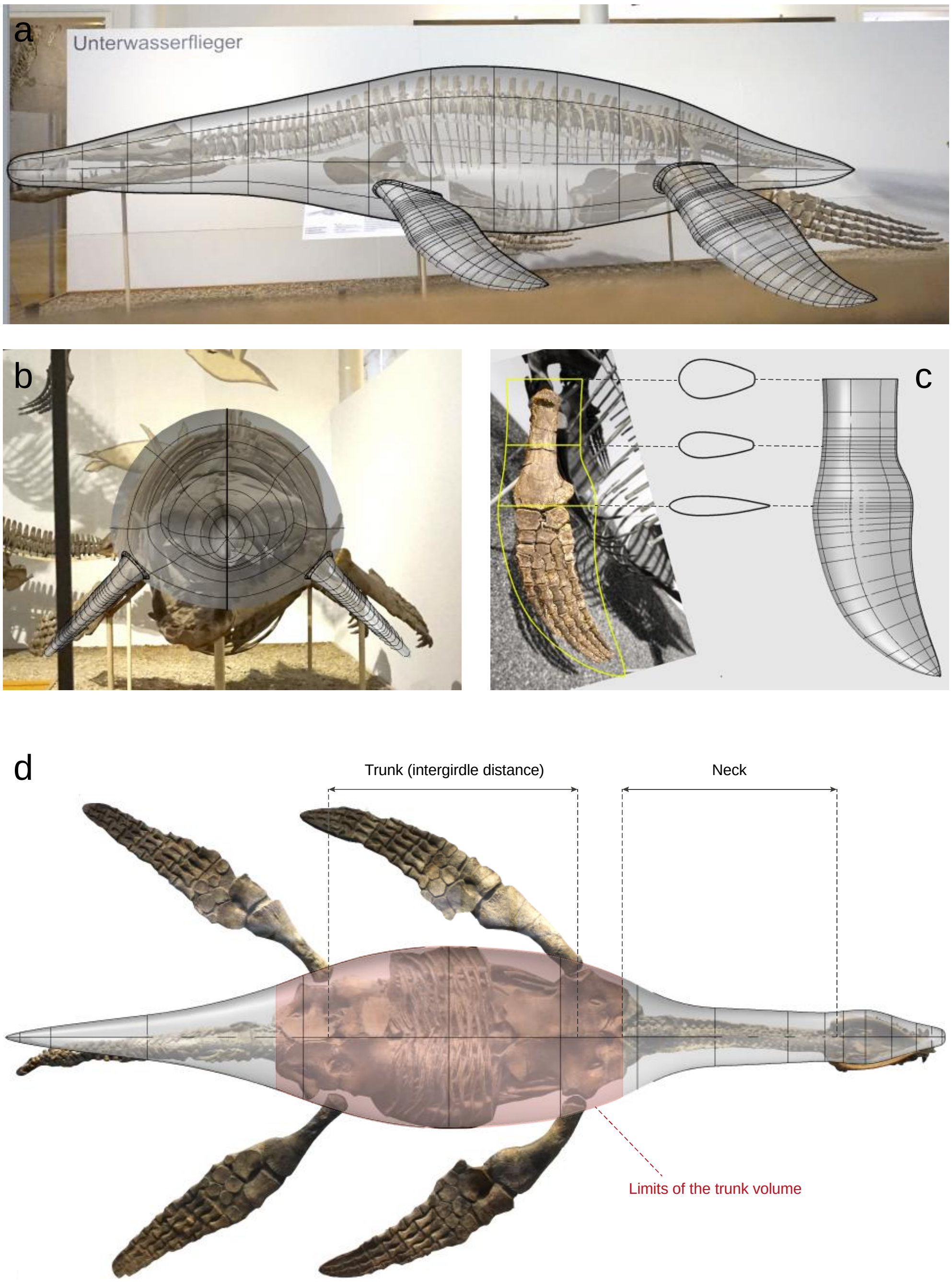

Supplementary Figure 8. 3D-modelling of fossil aquatic reptiles. NURBS model of the pliosaur *Liopleurodon ferox* in lateral view **(a)** and frontal view **(b)** showing the imported images of the fossil specimen (GPIT-RE-3184, palaeontological collection, University of Tübingen, Germany) used to outline the body contours in Rhinoceros v.5.0. **(c)** Modelling of the limbs, showing the limb bones in lateral view with the planform limits traced and the cross-section forms used to create a swept surface. (D) Model of the rhomaleosaur *Meyerasaurus victor*, a basal plesiosaur, overlaying the image of the fossil specimen used in the modelling process (SMNS 12478, State Museum of Natural History, Stuttgart, Germany). The intergirdle distance (trunk), neck length used to calculate the neck ratio (neck/trunk) are shown. Versions of this model with varying neck ratios were used to test the effect of neck on the drag. The trunk volume, which includes the girdles, is highlighted in red.

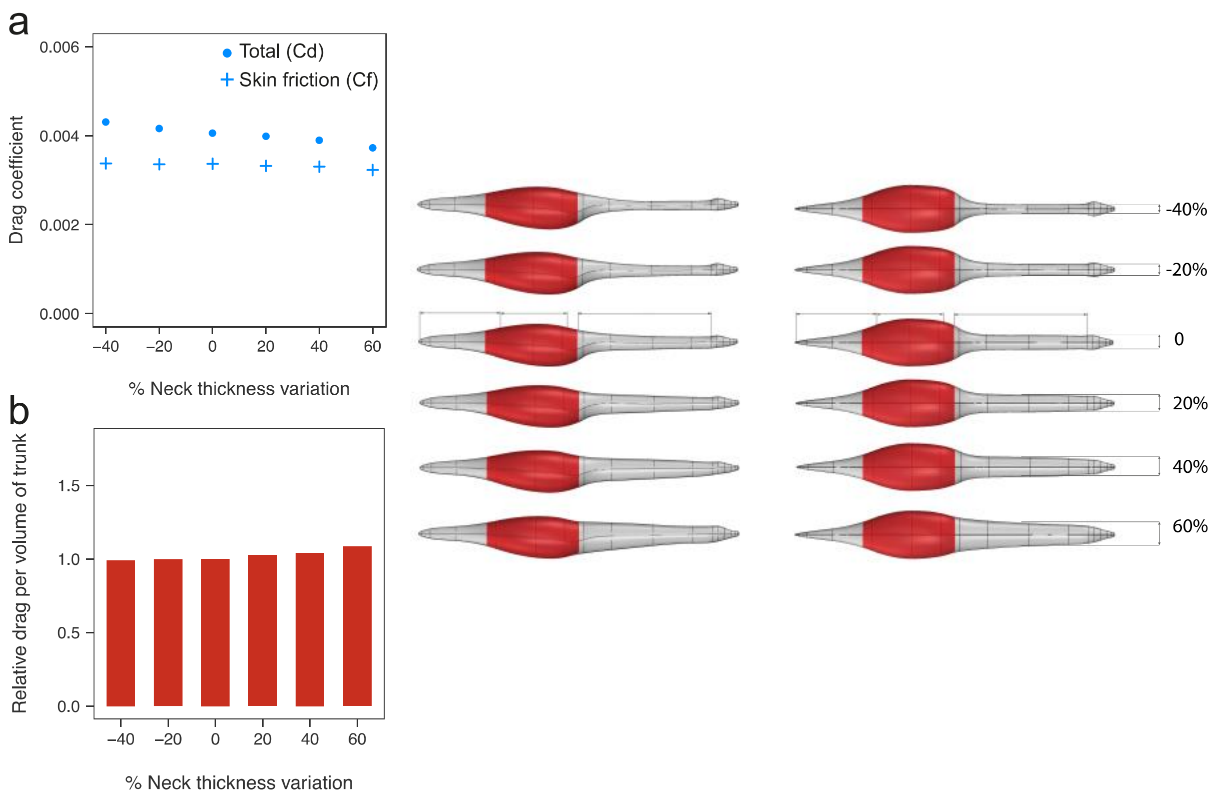

Supplementary Figure 9. **Sensitivity test for neck thickness.** Effect of varying neck thicknesses in the total and skin friction drag coefficient **(a)** and in the drag per unit of trunk volume **(b)** calculated on a hypothetical plesiosaur model with a neck ratio of 2×. The limits of the trunk in the models are highlighted in red. The variation in neck thickness is shown as a percentage of the width measured at the mid-point of the neck, relative to that in the original model.

Supplementary tables

	Taxon	L (m)	D (m)	FR	S (m^2)	V (m^3)	% Limb SA / S
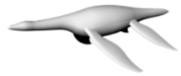	*Meyerasaurus*	1	0.193	5.18	0.464	0.0101	35.9
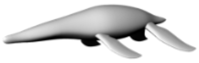	*Liopleurodon*	1	0.220	4.53	0.551	0.0153	30.9
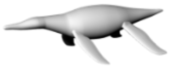	*Peloneustes*	1	0.215	4.65	0.486	0.0120	28.9
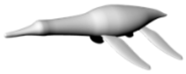	*Dolichorhynchops*	1	0.201	4.98	0.477	0.0111	32.3
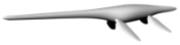	*Hydrotherosaurus*	1	0.115	8.70	0.206	0.0028	23.9
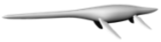	*Thalassomedon*	1	0.127	7.87	0.223	0.0037	18.3
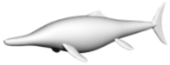	*Temnodontosaurus*	1	0.174	5.75	0.316	0.0073	9.8
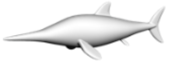	*Stenopterygius*	1	0.175	5.71	0.358	0.0095	10.0
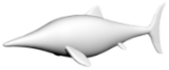	*Ophthalmosaurus*	1	0.213	4.69	0.387	0.0107	9.7
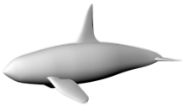	*Orcinus*	1	0.211	4.74	0.518	0.0156	12.0
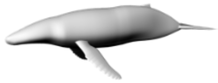	*Megaptera*	1	0.196	5.10	0.474	0.0134	12.0
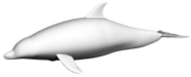	*Tursiops*	1	0.206	4.85	0.407	0.0116	6.7

Supplementary Table 1. Geometric parameters of the reconstructed taxa scaled to the same total length. Dimensions for an array of plesiosaur, ichthyosaur and modern cetacean models scaled to a full length of 1m. L is total body length, measured from the tip of the rostrum to the tip of the tail; D is the maximum diameter perpendicular to the longitudinal axis; FR is the fineness ratio calculated as L/D; S is the total surface area; V is the total body volume and % Limb SA / S is the percentage of surface area of limbs in relation to the total body surface. Dimensions were measured in Rhinoceros v.5.0.

	Comparison 1		Comparison 2		Comparison 3	
	Liopleurodon	***Tursiops***	***Liopleurodon***	***Tursiops***	***Liopleurodon***	***Tursiops***
	**Freshwater**	**Freshwater**	**Sea water**	**Sea water**	**Sea water**	**Sea water**
fluid density (ρ), Kg/m^3	998.2	998.2	1025	1025	1025	1025
fluid dynamic viscosity (μ), Ns/m^2	0.001002	0.001002	0.00109	0.00109	0.00109	0.00109
flow velocity (u), ms^{-1}	1	1	1.029	1.029	1	1
body length (l), m	4.88	2.85	4.88	2.85	4.88	2.85
body Volume (V), m^3	1.78	0.27	1.78	0.27	1.78	0.27
Reynolds number (*Re*)	4.9×10^6	2.8×10^6	4.9×10^6	2.8×10^6	4.6×10^6	2.7×10^6
Drag (D), N	35.06	8.36	35.06	8.36	31.03	7.37
Drag coefficient (C_d)	0.005355	0.005074	0.005355	0.005074	0.005399	0.005018
Drag/Volume (D/V), N/m^3	19.66	31.13	19.66	31.13	17.40	27.44
**Relative Drag/Volume**	**0.63**	**1**	**0.63**	**1**	**0.63**	**1**

Supplementary Table 2. Sensitivity test for the flow parameters. Comparisons the relative drag per unit of volume for the models *Liopleurodon* and *Tursiops* at life-size scale simulated at the same inlet velocity, applying two flow settings: freshwater and sea water at 20°C (see also Supplementary Data).

**Comparisons per group**	**Pairwise *t*-tests *p*-value**	
	**Same mass, same speed**	**Real scale, same speed**
Derived plesiosaurs / Parvipelvian ichthyosaurs	**1.33×10^{-5}**	0.676
Derived plesiosaurs / Modern cetaceans	**8.02×10^{-5}**	0.379
Parvipelvian ichthyosaurs / Modern cetaceans	0.623	0.388
Short-necked plesiosaurs (pliosauromorph) / Parvipelvian ichthyosaurs	**4.65×10^{-5}**	0.978
Short-necked plesiosaurs (pliosauromorph) / Modern cetaceans	**0.0008**	0.263

Supplementary Table 3. Comparative tests for differences in COT_{drag} for an array of plesiosaurs, parvipelvian ichthyosaurs and modern cetaceans. *p*-values of pairwise *t*-tests comparing the drag-related costs of steady swimming (COT_{drag}) when size is removed (same mass, same inlet velocity; as in Fig. 2a) and when body size is accounted for (real scale, same inlet velocity; as in Fig. 2b) between different groups (see also Supplementary Data). Numbers in bold denote statistical significance (p-value <0.05).

Comparisons per group	Pairwise *t*-tests *p*-value	Significance
Basal / Derived	0.053	NS
Short-necked (pliosauromorph) / Long-necked (plesiosauromorph)	0.399	NS
Short-necked (pliosauromorph) excluding basal / Long-necked (plesiosauromorph)	0.823	NS
Thalassophonea / Elasmosauridae	0.949	NS
Thalassophonea / Elasmosaurinae	0.886	NS

Supplementary Table 4. Comparative tests for differences in the drag per unit of trunk volume for plesiosaur groups. *p*-values of pairwise *t*-tests comparing the values of relative drag per unit of trunk volume when life-size scale is accounted for (real scale, same inlet velocity; as in Fig. 4e) between different plesiosaur groups and morphotypes (see also Supplementary Data).

Analysis	*n* trees log Bayes Factor of > 5, homogeneous vs heterogeneous rates	*n* trees log Bayes Factor of > 10, homogeneous vs heterogeneous rates
Cal3-dated trees, trunk	20/20, smallest log BF is 48.81584	20/20, smallest log BF is 48.81584
Cal3-dated trees, log_{10} trunk	20/20, smallest log BF is 12.34111	20/20, smallest log BF is 12.34111
Hedman-dated trees, trunk	20/20, smallest log BF is 6.020636	14/20, smallest log BF is 6.020636
Hedman-dated trees, log_{10} trunk	20/20, smallest log BF is 40.27451	20/20, smallest log BF is 40.27451
Cal3-dated trees, neck ratio	20/20, smallest log BF is 7.022984	18/20, smallest log BF is 7.022984
Hedman-dated trees, neck ratio	20/20, smallest log BF is 5.530324	0/20, smallest log BF is 5.530324

Supplementary Table 5. Proportion of analytical iterations favouring heterogeneous rates over homogeneous rates across all analyses. All iterations have log Bayes Factor of > 5 in support of heterogeneous rates.

	Tree number	Elasmosauridae	Polycotylidae	Thalassophonea
Trunk	1	**0.0396**	0.5992	0.9098
	2	0.599	0.334	**0.0199**
	3	**0.0169**	0.7322	0.9818
	4	0.892	0.8404	0.6971
	5	**0.0094**	0.2464	0.8754
	6	0.0854	0.6984	0.8023
	7	0.6686	0.7332	0.1232
	8	0.0932	0.995	0.1277
	9	0.2621	0.4342	0.8001
	10	**0.0027**	0.8075	0.8761
	11	**0.0185**	0.8343	0.3764
	12	0.7763	0.9889	0.8914
	13	0.1009	0.7737	0.0963
	14	0.055	0.8495	0.8948
	15	0.1058	0.5676	0.5807
	16	0.3547	0.8732	0.7419
	17	**0.0121**	0.9888	0.8956
	18	0.7434	**0.0417**	0.8183
	19	**0.0226**	0.4032	0.3103
	20	**0.0286**	0.2345	0.2332
Neck	1	**0.0019**	0.4568	0.7878
	2	**0.0001**	0.5299	0.8399
	3	**0.0001**	0.8243	0.4719
	4	**0.0003**	0.216	0.1766
	5	**0.0001**	0.8812	0.6028
	6	**0.0001**	0.1046	0.3905
	7	**0.0042**	0.8599	0.7095
	8	**0.0001**	0.1998	0.5005
	9	**0.0001**	0.995	0.7183
	10	**0.0001**	0.7651	0.3447
	11	**0.0001**	0.4978	0.9031
	12	**0.0125**	0.2173	0.8669
	13	**0.0001**	0.8193	0.3766
	14	**0.0001**	0.471	0.9185
	15	**0.0001**	0.2285	0.7633
	16	0.115	0.9941	0.5639
	17	**0.0001**	0.9868	0.4332
	18	0.1538	**0.0001**	0.7004
	19	**0.0001**	0.8225	0.5194
	20	**0.0001**	0.7882	0.4689

Supplementary Table 6. *p*-values for the randomization tests of trunk length and neck ratio rates of evolution in three clades of derived plesiosaurs. Results obtained from twenty cal3-dated phylogenies of Sauropterygia (see also Supplementary Figure 5). Values in bold indicate evolutionary rates significantly different from the background rate (*p*-value <0.05).

Supplementary methods

NURBS modelling

Illustrations and photographs were imported into Rhinoceros v. 5.0 to be used as background images. The outline of the body was traced on the lateral or dorsal view and rotated about the x-axis to create an axisymmetric body, which was further adjusted to match the width and depth of the body in the various views (Supplementary Fig. 6a, b). For plesiosaurs, the body outlines were created tapering smoothly around the neck and towards the tip of the tail as it is observed in the holotype of the polycotylid *Mauriciosaurus fernandezi*, a plesiosaur specimen that preserves a skin body impression in ventral view[1]. *Dolichorhynchops*, *Liopleurodon*, *Peloneustes* and *Thalassomedon* are based on specimens mounted in 3D (KUVP 1300, GPIT-RE-3184, GPIT-RE-3182 and DMNH 1588 respectively) and thus the body outline could be adjusted to dorsal, lateral and frontal views (Supplementary Fig. 6a, b). *Meyerasaurus* was based on a complete specimen that was only exposed on ventral view (SMNS 12478), therefore the lateral body width (i.e. the width of the ribcage) for this model was based on other plesiosaur specimens that preserve this information[2], as done in a previous 3D reconstruction of *Meyerasaurus*[3]. The model for *Hydrotherosaurus* was based on the full-body reconstruction in illustrated in lateral view, the reconstructed pelvic and pectoral cross-sections and detailed photographic material by Welles[4]. Ichthyosaurs were modelled as described in Gutarra et al., 2019[5]. All three ichthyosaur taxa considered in this analysis are members of the parvipelvian clade. Based on specimens of exceptional preservation for this group (e.g. *Stenopterygius*[6], *Hauffiopteryx*[7], *Aegirosaurus*[8]), the most reasonable assumption is that they possessed dorsal fins and lunate caudal flukes, which we followed to model the taxa with no soft tissue preservation (i.e. *Temnodontosaurus* and *Ophthamosaurus*). Because these elements are tapered and highly streamlined, variations in shape and dimensions have a very small impact on drag when oriented horizontally to the flow[9].

The limbs of fossil ichthyosaurs and modern cetaceans were modelled using the sweep-two rail function as shown elsewhere[5]. In the plesiosaur specimens, the planform outlines were traced around the limb bones in lateral view and three cross-sections were placed along the length of the limb: a fusiform streamlined profile of 20% maximum thickness (i.e. maximum diameter divided by the chord length) at the distal end of the propodial bone, and two thicker profiles, one halfway and another at the base of the propodium, to adapt to the areas where these bones become wider (Supplementary Fig. 6c). The cross-sections and planform outlines served to create the limb geometry using the sweep-two rail function. The limbs were oriented at about 45 degrees relative to the longitudinal body axis in a feathered position (i.e. at a zero angle of attack relative to the flow), as previously modelled elsewhere[5] and consistent with gliding in extant cetaceans. Such configuration results in minimum drag for the limbs and aids in attaining a lift close to zero in simulations that include the limbs. The limbs were joined to the body using a Boolean function and a curved fillet edge was created to soften the flipper insertion in order to avoid interference drag due to sharp corners. The final geometries were scaled to a

total length of 1 m and exported in STP format. All models used in the present analysis are available for download (see Data availability).

Sensitivity tests

E*xternal flow parameters:*

The flow simulations presented herein have been performed using the default program settings for water, corresponding to freshwater at 20°C. The drag coefficient is a non-dimensional parameter, therefore, is independent of the flow density and viscosity. Our comparison of drag per unit of volume are also not affected by using a different density of water. To illustrate this, we performed additional simulations for two of the morphologies of this study (*Liopleurodon* and *Tursiops*) for the same reference velocity at life-size scales, using both freshwater and sea water conditions (Supplementary Table 2 and Supplementary Data). This table shows that the small difference of density and viscosity between the two types of water causes minor changes in the absolute values of drag but importantly, the comparisons of the relative drag per unit of volume, which are the focus of our research, remain unchanged.

Uncertainties in the morphology of fossil taxa:

The influence of modelling uncertainty in this CFD methodology has been tested by sensitivity analyses in a previous publication[5]. These sensitivity tests showed that various potential sources of uncertainty have a small effect on the drag coefficient over a wide range of variation: for example, increasing or decreasing the body width up to 30% results in ≤ 3 % variation of C_d at a Reynolds number of 10^7, and the thickness of limbs only impacts the C_d in less than 5% within a 40% variation[5].

Recently, a CFD-based study suggested that thick necks can decrease the drag coefficient and therefore provide a hydrodynamic advantage[10]. The effect of this anatomical characteristic in the present results was assessed through a sensitivity test, where various parametrized neck thicknesses were applied to the model with a neck ratio of 2× (Supplementary Fig. 9). We created variations of this model in which the neck thickness, as measured halfway the neck length, was decreased or increased by 20% intervals resulting in thinner and thicker neck profiles. A reduction of up to 3.9% and 8.1% is observed in the total drag coefficient for necks that are 40% and 60% thicker than the original model, but there was no reduction in the proportion of drag per unit of trunk volume. On the contrary, this parameter was 4.1% and 8.4% higher for necks 40% and 60% thicker than the original model.

Composite Sauropterygian Tree

Elasmosaurid relationships were updated according to a recently published phylogeny of elasmosaurs[11]. The following new sauropterygian taxa published in recent years were added to the composite tree, *Atopodentatus unicus*[12], *Dianmeisaurus gracilis*[13], *Lindwurmia thiuda*[14], *Lushkan itilensis*[15], *Panzhousaurus rotundirostris*[16], *Sachicasaurus vitae*[17] and *Wangosaurus brevirostris*[18]. The first and

last appearances (FAD and LAD) of each species were based on the age limits of the geological formations where fossils were found, according to their references (Supplementary Data).

Time scaling

The phylogeny was time-calibrated using two methods to account for dating uncertainty, the cal3[19,20] method and the Hedman method[21,22]. The cal3 method is a probabilistic time-scaling approach implemented using the bin_cal3TimePaleoPhy function from the paleotree R package[19]. The method samples divergence times under a birth-death model, with a priori inputs of branching, extinction, and sampling rates[19,20,22,23]. We used published rates from the literature of the tetrapod fossil record, with instantaneous sampling rates uniformly sampled ranging from 0.042 lmy^{-1} to 0.18 lmy^{-1} [22–25]. From this, we then calculated the extinction and origination rates. Zero-length branches were replaced with 0.0001-million-year branch lengths to avoid analytical issues downstream. The 'Hedman' dating method is another widely applied Bayesian approach that dates nodes using probability distributions constrained by successive outgroup taxon ages[13]. We use the 'whole-tree extension' of the Hedman algorithm[14]. Occurrence dates for successive outgroup taxa that predated Sauropterygia and each subsequent outgroup were required, we used: *Microcnemus efremovi* (251.2–249.2 Ma), *Claudiosaurus germaini* (256.62–251.902 Ma), *Youngina capensis* (259.1–251.902 Ma), *Lanthanolania ivakhnenkoi* (272.95-265.1Ma), *Araeoscelis gracilis* (283.5–278.225 Ma), and *Petrolacosaurus kansensis* (307–303.7 Ma).

Supplementary references

1. Frey, E. *et al.* A new polycotylid plesiosaur with extensive soft tissue preservation from the early Late Cretaceous of northeast Mexico. *Boletín de la Sociedad Geológica Mexicana* **69**, 87–134 (2017).
2. O'Keefe, F. R., Street, H. P., Wilhelm, B. C., Richards, C. D. & Zhu, H. A new skeleton of the cryptoclidid plesiosaur *Tatenectes laramiensis* reveals a novel body shape among plesiosaurs. *Jounal of Vertebrate Paleontology* **31**, 330–339.
3. Liu, S. *et al.* Computer simulations imply forelimb-dominated underwater flight in plesiosaurs. *PLOS Computational Biology* **11**, e1004605 (2015).
4. Welles, S. P. Elasmosaurid plesiosaurs with description of new material from California and Colorado. *Memoirs of the University of California* **13**, 125–254 (1943).
5. Gutarra, S. *et al.* Effects of body plan evolution on the hydrodynamic drag and energy requirements of swimming in ichthyosaurs. *Proceedings of the Royal Society B: Biological Sciences* **286**, 20182786 (2019).
6. Maisch, M. W. Revision der gattung *Stenopterygius* Jaekel, 1904 emend. von Huene, 1922 (Reptilia: Ichthyosauria) aus dem unteren Jura Westeuropas. *Palaeodiversity* **1**, 227–271 (2008).

7. Maxwell, E. E. & Cortés, D. A revision of the Early Jurassic ichthyosaur <i>Hauffiopteryx<i> (Reptilia: Ichthyosauria), and description of a new species from southwestern Germany. *Palaeontol Electron* **23**, 1–43 (2020).
8. Bardet, N. & Fernandez, M. A new ichthyosaur from the Upper Jurassic lithographic limestones of Bavaria. *Journal of Paleontology* **74**, 503–511 (2000).
9. Hoerner, S. F. *Fluid-Dynamic Drag: Practical Information on Aerodynamic Drag and Hydrodynamic Resistance*. (Published by S.F. Hoerner, 1965).
10. Tröelsen, P. V., Wilkinson, D. M., Seddighi, M., Allanson, D. R. & Falkingham, P. L. Functional morphology and hydrodynamics of plesiosaur necks: does size matter? *Journal of Vertebrate Paleontology* **39**, e1594850 (2019).
11. O'Gorman, J. P. Elasmosaurid phylogeny and paleobiogeography, with a reappraisal of *Aphrosaurus furlongi* from the Maastrichtian of the Moreno Formation. *Journal of Vertebrate Paleontology* **39**, e1692025 (2019).
12. Cheng, L., Chen, X.-H., Shang, Q.-H. & Wu, X.-C. A new marine reptile from the Triassic of China, with a highly specialized feeding adaptation. *Naturwissenschaften* **101**, 251–259 (2014).
13. Shang, Q.-H., Li, C. & Wu, X.-C. New information on *Dianmeisaurus gracilis* Shang & Li, 2015. *Vertebrata PalAsiatica* **55**, 145–161 (2017).
14. Vincent, P. & Storrs, G. W. *Lindwurmia*, a new genus of Plesiosauria (Reptilia: Sauropterygia) from the earliest Jurassic of Halberstadt, northwest Germany. *Sci Nat* **106**, 1–18 (2019).
15. Fischer, V., Bardet, N., Benson, R. B. J., Arkhangelsky, M. S. & Friedman, M. Extinction of fish-shaped marine reptiles associated with reduced evolutionary rates and global environmental volatility. *Nature Communications* **7**, 10825 (2016).
16. Jiang, D.-Y., Lin, W.-B., Rieppel, O., Motani, R. & Sun, Z.-Y. A new Anisian (Middle Triassic) eosauropterygian (Reptilia, Sauropterygia) from Panzhou, Guizhou Province, China. *Journal of Vertebrate Paleontology* **38**, e1480113 (2018).
17. Páramo-Fonseca, M. E., Benavides-Cabra, C. D. & Gutiérrez, I. E. A new large pliosaurid from the Barremian (Lower Cretaceous) of Sáchica, Boyacá, Colombia. *Earth sci. res. j.* **22**, 223–238 (2018).
18. Ma, L.-T., Jiang, D.-Y., Rieppel, O., Motani, R. & Tintori, A. A new pistosauroid (Reptilia, Sauropterygia) from the late Ladinian Xingyi marine reptile level, southwestern China. *Journal of Vertebrate Paleontology* **35**, e881832 (2015).
19. Bapst, D. W. paleotree: an R package for paleontological and phylogenetic analyses of evolution: Analyses of Paleo-Trees in R. *Methods in Ecology and Evolution* **3**, 803–807 (2012).
20. Bapst, D. W. A stochastic rate-calibrated method for time-scaling phylogenies of fossil taxa. *Methods in Ecology and Evolution* **4**, 724–733 (2013).
21. Hedman, M. M. Constraints on clade ages from fossil outgroups. *Paleobiology* **36**, 16–31 (2010).

22. Bapst, D. W., Wright, A. M., Matzke, N. J. & Lloyd, G. T. Topology, divergence dates, and macroevolutionary inferences vary between different tip-dating approaches applied to fossil theropods (Dinosauria). *Biology Letters* **12**, 20160237 (2016).
23. Bapst, D. W. & Hopkins, M. J. Comparing cal3 and other a posteriori time-scaling approaches in a case study with the pterocephaliid trilobites. *Paleobiology* **43**, 49–67 (2017).
24. Friedman, M. & Brazeau, M. D. Sequences, stratigraphy and scenarios: what can we say about the fossil record of the earliest tetrapods? *Proceedings of the Royal Society B: Biological Sciences* **278**, 432–439 (2011).
25. Benson, R. B. J., Hunt, G., Carrano, M. T. & Campione, N. Cope's rule and the adaptive landscape of dinosaur body size evolution. *Palaeontology* **61**, 13–48 (2018).
