# Supplementary material for: Large size in aquatic tetrapods compensates for high drag caused by extreme body proportions
Source: Commun Biol. 2022 Apr 28;5:380. doi: 10.1038/s42003-022-03322-y (PMC9051157; doi:10.1038/s42003-022-03322-y)
Supplement: Supplementary file 3 — Description of Additional Supplementary Files [file 42003_2022_3322_MOESM3_ESM.pdf]

Description of Additional Supplementary Files

File name: Supplementary Data
Description: Calculations supporting the present results and datasets used in the evolutionary rates analyses.
