# Supplementary material for: Large size in aquatic tetrapods compensates for high drag caused by extreme body proportions
Source: Commun Biol. 2022 Apr 28;5:380. doi: 10.1038/s42003-022-03322-y (PMC9051157; doi:10.1038/s42003-022-03322-y)
Supplement: Supplementary file 5 — Reporting summary [file 42003_2022_3322_MOESM5_ESM.pdf]

nature research

Corresponding author(s): Susana Gutarra Diaz

Last updated by author(s): Mar 17, 2022

Reporting Summary

Nature Research wishes to improve the reproducibility of the work that we publish. This form provides structure for consistency and transparency in reporting. For further information on Nature Research policies, see our Editorial Policies and the Editorial Policy Checklist.

Statistics

For all statistical analyses, confirm that the following items are present in the figure legend, table legend, main text, or Methods section.

n/a	Confirmed	
☐	☒	The exact sample size (*n*) for each experimental group/condition, given as a discrete number and unit of measurement
☐	☒	A statement on whether measurements were taken from distinct samples or whether the same sample was measured repeatedly
☐	☒	The statistical test(s) used AND whether they are one- or two-sided<br>*Only common tests should be described solely by name; describe more complex techniques in the Methods section.*
☐	☒	A description of all covariates tested
☐	☒	A description of any assumptions or corrections, such as tests of normality and adjustment for multiple comparisons
☐	☒	A full description of the statistical parameters including central tendency (e.g. means) or other basic estimates (e.g. regression coefficient) AND variation (e.g. standard deviation) or associated estimates of uncertainty (e.g. confidence intervals)
☐	☒	For null hypothesis testing, the test statistic (e.g. *F*, *t*, *r*) with confidence intervals, effect sizes, degrees of freedom and *P* value noted<br>*Give P values as exact values whenever suitable.*
☐	☒	For Bayesian analysis, information on the choice of priors and Markov chain Monte Carlo settings
☒	☐	For hierarchical and complex designs, identification of the appropriate level for tests and full reporting of outcomes
☐	☒	Estimates of effect sizes (e.g. Cohen's *d*, Pearson's *r*), indicating how they were calculated

Our web collection on statistics for biologists contains articles on many of the points above.

Software and code

Policy information about availability of computer code

Data collection	No software was used for data collection.
Data analysis	The 3D-modelling software Rhino was used to create 3D models of ichtyosaurs, plesiosaurs and cetaceans (Academic v.5, https://www.rhino3d.com). All models used in this paper have been included in the supplementary information hosted at GitHub (https://github.com/SusanaGutarra/Plesiosaur-hydrodynamics-evolution) and can be found on Zenodo (https://doi.org/10.5281/zenodo.5979631).<br>The commercial software ANSYS-Fluent (Academic v.18.1) was used to perform the computer flow simulations.<br>Custom code in R was used in the tree time-scaling, ancestral state analysis, evolutionary rates analysis and clade randomisation tests.<br>BayesTraits (http://www.evolution.rdg.ac.uk/BayesTraitsV2.0.2.html) was used to test evolutionary rates. All code has been included in the supplementary information and is hosted at GitHub (https://github.com/SusanaGutarra/Plesiosaur-hydrodynamics-evolution) and can be found on Zenodo (https://doi.org/10.5281/zenodo.5979631).

For manuscripts utilizing custom algorithms or software that are central to the research but not yet described in published literature, software must be made available to editors and reviewers. We strongly encourage code deposition in a community repository (e.g. GitHub). See the Nature Research guidelines for submitting code & software for further information.

Data

Policy information about availability of data

All manuscripts must include a data availability statement. This statement should provide the following information, where applicable:

- Accession codes, unique identifiers, or web links for publicly available datasets
- A list of figures that have associated raw data
- A description of any restrictions on data availability

Supplementary Information includes Supplementary Figures, Supplementary Tables and Supplementary Methods; Supplementary Data includes calculations

supporting the present results and datasets used in the evolutionary rates analyses. These files, as well as the digital models created for the computer flow simulations performed in this study, can be found at https://doi.org/10.5281/zenodo.5979631.

Field-specific reporting

Please select the one below that is the best fit for your research. If you are not sure, read the appropriate sections before making your selection.

☐ Life sciences ☐ Behavioural & social sciences ☒ Ecological, evolutionary & environmental sciences

For a reference copy of the document with all sections, see nature.com/documents/nr-reporting-summary-flat.pdf

Ecological, evolutionary & environmental sciences study design

All studies must disclose on these points even when the disclosure is negative.

Study description	We study the effect of morphology and size on the hydrodynamic performance of large aquatic tetrapods using 3D modelling and computer flow simulations in plesiosaurs, ichthyosaurs and modern cetaceans. We then test how the neck length impacted the drag and swimming performance of plesiosaurs, and whether these effects are dependent on body size. Finally, we study the evolution of neck proportions and body size in this group using evolutionary rates analyses, and discuss the implications for swimming performance.
Research sample	For the computational fluid dynamics (CFD) analyses, we used full-body 3D models of 6 plesiosaurs (Meyerasaurus victor, Liopleurodon ferox, Peloneustes phylarchus, Dolichorhynchops osborni, Hydrotherosaurus alexandrae and Thalassomedon hanningtoni); 3 ichthyosaurs (Temnodontosaurus platyodon, Stenopterygius quadriscissus and Ophthalmosaurus icenicus) and 3 extant cetaceans (Tursiops truncatus, Orcinus orca and Megaptera novaengliae).<br>Additionally, for CFD analysis focusing on interplay of neck length and trunk size, we added the limbless models of 7 additional taxa to the 6 above mentioned plesiosaurs (Rhomaleosaurus thorntoni, Plesiosaurus sp., Polycotylus latippinus, Kronosaurus boyacensis, Styxosaurus sp, Albertonectes vanderveldei and Aristonectes quiriquinensis).<br>The evolutionary analysis of neck proportions and body size are based on a dataset of 79 sauropterygian species, with data collected from personal observation and the literature (see Supplementary Information for the detail on the sources).
Sampling strategy	The criterion for including fossil taxa in the CFD analysis was completeness. All fossils used to create 3D full-body models preserve the greatest part of the axial skeleton and the limbs, some of them are preserved and mounted in 3D or have been described and figured in detail in the literature. The limbless models added for the CFD analysis of trunk and neck are based on completeness of the axial skeleton (head, neck, trunk and tail).<br>79 sauropterygian taxa were included in the analysis of evolutionary rates based on completeness of neck and trunk.
Data collection	The 3D models are based on photographs or reconstructions from the literature and direct observation where possible.<br>Continuous measurements of the trunk and length were taken from the primary literature or measurements by SG using measuring tape (see Supplementary Information for the detail on the sources). The sauropterygian composite tree was modified from the one used in Stubbs & Benton, 2016 including recently described taxa. Occurrence data was collected by SG from published records, based on the limits of the geological formations where fossils are found.
Timing and spatial scale	The 3D model creation, data collection and analyses were performed over a 3-year period, including visits to museums in the UK, Germany, Italy and Switzerland.
Data exclusions	N/A
Reproducibility	For the CFD analyses, a detailed account of the meshing strategy, dimensions of the fluid domain, physics, turbulence model, boundary conditions and solver parameters is provided in the methods which will allow reproducing the simulations in ANSYS-Fluent with the 3D models that are available for download in https://doi.org/10.5281/zenodo.5979631.<br>All measurements, phylogenetic tree and R code for the evolutionary analyses is included in the Supplementary information (https://doi.org/10.5281/zenodo.5979631).
Randomization	Differences in evolutionary rates between plesiosaur groups were tested using a randomisation test, consisting in performing multiple random samplings of mean rate values and calculating differences between sampled and non-sampled branches over 9999 replicates.
Blinding	N/A

Did the study involve field work? ☐ Yes ☒ No

Reporting for specific materials, systems and methods

We require information from authors about some types of materials, experimental systems and methods used in many studies. Here, indicate whether each material, system or method listed is relevant to your study. If you are not sure if a list item applies to your research, read the appropriate section before selecting a response.

Materials & experimental systems

n/a	Involved in the study
☒	☐ Antibodies
☒	☐ Eukaryotic cell lines
☐	☒ Palaeontology and archaeology
☒	☐ Animals and other organisms
☒	☐ Human research participants
☒	☐ Clinical data
☒	☐ Dual use research of concern

Methods

n/a	Involved in the study
☒	☐ ChIP-seq
☒	☐ Flow cytometry
☒	☐ MRI-based neuroimaging

Palaeontology and Archaeology

Specimen provenance

All specimens that served to create 3D models or as a source of measurements come from internationally recognised institutions, and were visited by SG with the permission of the curators, or their photographs and reconstructions were sourced from published scientific literature (see Supplementary Information for the detail on specimen numbers and references).

Specimen deposition

Specimen data were collected from the literature or personal observation (see Supplementary Information for the detail on specimen numbers and sources of the data).

Dating methods

Occurrence data was collected by SG from published records, based on the limits of the geological formations where fossils are found.

☐ Tick this box to confirm that the raw and calibrated dates are available in the paper or in Supplementary Information.

Ethics oversight

N/A

Note that full information on the approval of the study protocol must also be provided in the manuscript.
